# A New Mouse Model of Chronic Myocarditis Induced by Recombinant Bacille Calmette–Guèrin Expressing a T-Cell Epitope of Cardiac Myosin Heavy Chain-α

**DOI:** 10.3390/ijms22020794

**Published:** 2021-01-14

**Authors:** Kazuko Tajiri, Kyoko Imanaka-Yoshida, Yusuke Tsujimura, Kazuhiro Matsuo, Michiaki Hiroe, Kazutaka Aonuma, Masaki Ieda, Yasuhiro Yasutomi

**Affiliations:** 1 Tsukuba Primate Research Center, National Institutes of Biomedical Innovation, Health and Nutrition, Tsukuba 305-0843, Japan; tsujim-y@niid.go.jp; 2 Department of Cardiology, Faculty of Medicine, University of Tsukuba, Tsukuba 305-8575, Japan; kaonuma@md.tsukuba.ac.jp (K.A.); mieda@md.tsukuba.ac.jp (M.I.); 3 Department of Pathology and Matrix Biology, Mie University Graduate School of Medicine, Tsu 514-8507, Japan; imanaka@doc.medic.mie-u.ac.jp; 4 Mie University Matrix Biology Research Center, Mie University Graduate School of Medicine, Tsu 514-8507, Japan; 5 Leprosy Research Center, National Institute of Infectious Diseases, Higashimurayama 189-0002, Japan; 6 Department of Research and Development, Japan BCG Laboratory, Kiyose 204-0022, Japan; matsuo@bcg.gr.jp; 7 Department of Cardiology, National Center for Global Health and Medicine, Tokyo 162-8655, Japan; hiroem55@yahoo.co.jp

**Keywords:** inflammatory dilated cardiomyopathy, myocarditis, BCG, recombinant BCG, autoimmunity, autoimmune myocarditis

## Abstract

Dilated cardiomyopathy (DCM) is a potentially lethal disorder characterized by progressive impairment of cardiac function. Chronic myocarditis has long been hypothesized to be one of the causes of DCM. However, owing to the lack of suitable animal models of chronic myocarditis, its pathophysiology remains unclear. Here, we report a novel mouse model of chronic myocarditis induced by recombinant bacille Calmette-Guérin (rBCG) expressing a CD4^+^ T-cell epitope of cardiac myosin heavy chain-α (rBCG-MyHCα). Mice immunized with rBCG-MyHCα developed chronic myocarditis, and echocardiography revealed dilation and impaired contraction of ventricles, similar to those observed in human DCM. In the heart, CD62L^−^CD4^+^ T cells were increased and produced significant amounts of IFN-γ and IL-17 in response to cardiac myosin. Adoptive transfer of CD62L^−^CD4^+^ T cells induced myocarditis in the recipient mice, which indicated that CD62L^−^CD4^+^ T cells were the effector cells in this model. rBCG-MyHCα-infected dendritic cells produced proinflammatory cytokines and induced MyHCα-specific T-cell proliferation and Th1 and Th17 polarization. This novel chronic myocarditis mouse model may allow the identification of the central pathophysiological and immunological processes involved in the progression to DCM.

## 1. Introduction

Dilated cardiomyopathy (DCM) is a heterogeneous group of myocardial diseases. Myocarditis has long been hypothesized as one of the causes of DCM [1]. Myocarditis is an inflammatory disease of the myocardium that is generally self-limited. However, in several cases, prolonged inflammation eventually results in DCM, which is a potentially lethal disorder characterized by progressively impaired cardiac function [2,3]. Long-term follow-up studies in patients with acute myocarditis have documented the development of DCM in 21% of patients over a mean follow-up period of 3 years [4]. Despite ongoing advances in the treatment of heart failure, improvement of DCM outcome remains problematic. Therefore, it is important to elucidate the molecular basis of the immune processes involved in the transition from myocarditis to DCM.

Myocarditis can be triggered by many different environmental agents, including viral and bacterial infections, toxins, and drugs [5,6], and subsequent autoimmune response is thought to contribute to the disease progression to DCM [7,8]. Among several cardiac self-antigens targeted during chronic heart inflammation, cardiac myosin heavy chain α isoform (MyHCα) has been identified as the most prominent autoantigen in myocarditis and DCM [9,10]. MyHCα immunization with immune adjuvants or the injection of MyHCα-loaded dendritic cells (DCs) can induce autoimmune myocarditis in mice [11,12]. In these models, myocardial inflammation persists for only 2–3 weeks [8,12,13]; therefore, a chronic autoimmune myocarditis model is needed to uncover the mechanisms of progression from myocarditis to DCM.

Bacille Calmette-Guérin (BCG), a live attenuated vaccine derived from *Mycobacterium bovis*, is the only licensed vaccine that has substantially helped controlling tuberculosis. Recombinant BCG (rBCG), which expresses foreign antigens or molecules, has been attracting attention as a promising vaccine candidate to protect against infectious diseases (such as tuberculosis and acquired immune deficiency syndrome) and cancers [14]. Since BCG is stable and highly immunogenic, possesses an inherent adjuvant capacity, and replicates inside macrophages and DCs [15], antigens expressed by rBCG can elicit long-lasting humoral and cellular immunity, including a CD4^+^ T-cell response to the recombinant antigen [16,17]. Therefore, we hypothesized that rBCG designed to express a cardiac autoantigen could elicit autoantigen-specific immune responses on a long-term basis, inducing chronic myocarditis and DCM.

Here, we report a novel mouse model of chronic myocarditis induced by rBCG expressing a cardiac autoantigen. Immunization with an rBCG expressing a CD4^+^ T-cell epitope of MyHCα (rBCG-MyHCα) elicited MyHCα-specific T-cell responses on a long-term basis and induced chronic myocarditis with dilation and impaired contraction of heart ventricles similar to human DCM. 

## 2. Results

### 2.1. Characterization of rBCG-MyHCα

**Figure 1:**
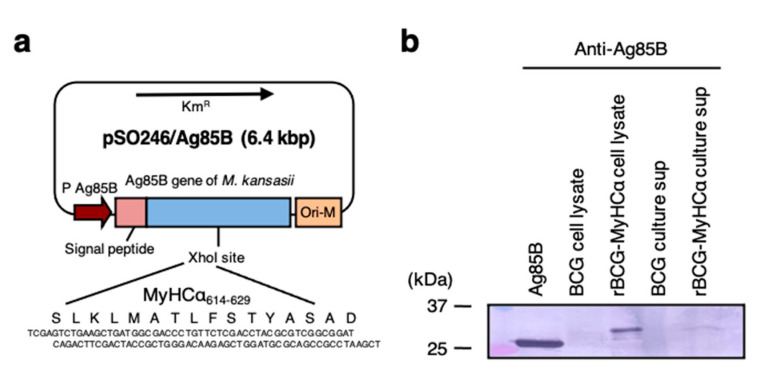
Construction and characterization of rBCG-MyHCα. (**a**) Oligonucleotides that encode a MyHCα CD4 epitope were cloned into an XhoI site in the coding region of the Ag85B gene from *Mycobacterium kansasii* cloned into pSO246 to be fused with the gene, and the plasmid was introduced into BCG by electroporation, giving rise to rBCG-MyHCα. (**b**) Western blot analysis of recombinant Ag85 protein or *M. kansasii*, cell lysates of BCG and rBCG-MyHCα, and culture supernatants of BCG and rBCG-MyHCα.

To enhance immunogenicity, we constructed rBCG-MyHCα that could express a CD4^+^ T-cell epitope of MyHCα as a fusion protein with Ag85B from *Mycobacterium kansasii*, a mycobacterial antigen known to induce strong T helper (Th)1 and Th17 type immune responses in the host [18,19,20]. The construction of the plasmid used for expression of the MyHCα-Ag85B fusion protein is shown in Figure 1a. For the generation of rBCG-MyHCα and rBCG-pSO246 (control), BCG was transformed with pSO246-MyHCα and a pSO246 empty vector, respectively. Western blot analyses of rBCG-MyHCα cell lysates and their culture supernatants revealed that rBCG-MyHCα produced MyHCα-Ag85B fusion protein in the bacteria and also secreted it stably in the culture supernatant (Figure 1b). 

### 2.2. rBCG-MyHCα Immunization Induces Chronic Myocarditis and DCM in Mice

**Figure 2:**
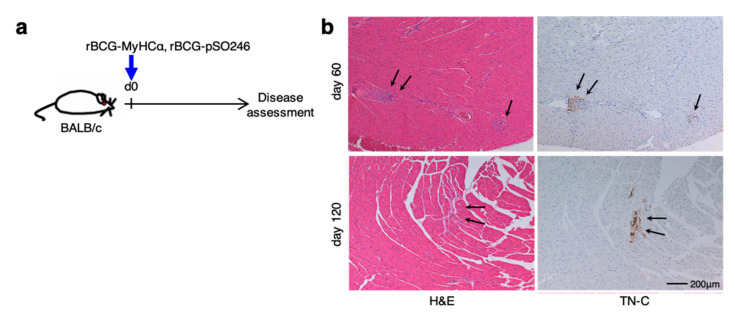
A single injection of rBCG-MyHCα induced chronic but weak myocarditis. (**a**) Mice were immunized with rBCG-MyHCα or rBCG-pSO246 on day 0. (**b**) Representative histology (H&E staining and TN-C immunostaining) of the heart sections on days 60 and 120 after rBCG immunization. Arrows indicate myocardial inflammation.

Next, we investigated the ability of rBCG-MyHCα to induce chronic myocarditis in vivo. rBCG-MyHCα induced a small cluster of mononuclear cell infiltration with the expression of tenascin-C (TN-C), a marker of active inflammation [21] (Figure 2), and a prevalence of 35.7% (5/14). 

**Figure 3:**
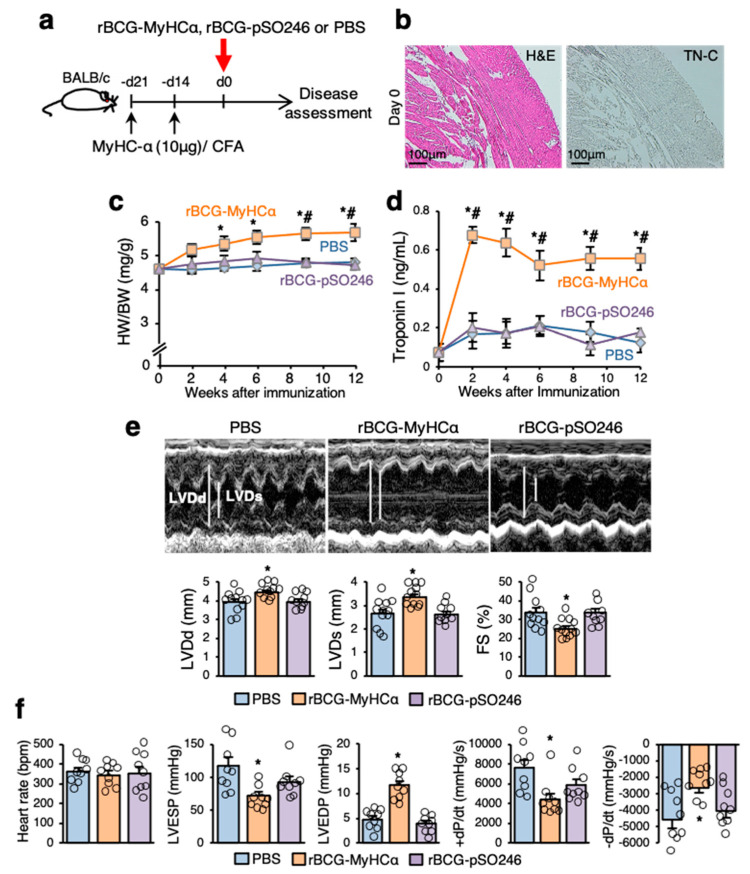
rBCG-MyHCα immunization induced heart failure in mice. (**a**) Mice were immunized twice with 10 μg of MyHCα peptide/CFA on days 14 and 21 before rBCG immunization for priming. rBCG-MyHCα, rBCG-pSO246, or PBS were injected subcutaneously on day 0. (**b**) Representative histology (H&E staining and TN-C immunostaining) of the heart sections on day 0 (21 days after first immunization with MyHCα). (**c**,**d**) HW/BW (**c**) and serum troponin I concentrations (**d**) at indicated time points are shown (*n* = 10 each). Results are presented as mean ± SEM. * *p* < 0.05 vs. PBS and # *p* < 0.05 vs. rBCG-pSO246 by one-way ANOVA with Tukey’s post hoc test. (**e**) Representative M mode images of echocardiography 12 weeks after rBCG immunization. Bar graphs represent echocardiographic parameters. Results are presented as mean ± SEM, *n* = 9–12, * *p* < 0.05 vs. PBS and rBCG-pSO246 by Kruskal-Wallis analysis with a post hoc Steel-Dwass test. (**f**) Bar graphs represent hemodynamic parameters at 12 weeks after rBCG immunization. Results are presented as mean ± SEM, *n* = 9 each, * *p* < 0.05 vs. PBS and rBCG-pSO246 by Kruskal-Wallis analysis with a post hoc Steel-Dwass test.

Thus, a single injection of rBCG-MyHCα induced mild chronic myocarditis at low prevalence. Therefore, to boost the immune response, mice were immunized with a small amount of MyHCα peptide before rBCG-MyHCα immunization (Figure 3a). As shown in Figure 3b, such a small amount of MyHCα peptide did not induce myocardial inflammation in mice, as previously reported [12]. The rBCG-MyHCα-immunized mice primed with small amounts of MyHCα peptides conjugated with complete Freund’s adjuvant (CFA) showed progressively increased heart-to-body weight ratios (HW/BW) and elevated serum levels of troponin I, a marker of cardiomyocyte damage, for more than 12 weeks (Figure 3c,d). Echocardiographic examination revealed an acceleration of cardiac dilatation and deterioration of left ventricular (LV) function in rBCG-MyHCα-immunized mice (Figure 3e). In cardiac catheterization experiments, rBCG-MyHCα-immunized mice showed lower LV end-systolic pressure (LVESP), higher LV end-diastolic pressure (LVEDP), and lower maximum and minimum dP/dt (±dP/dT) (Figure 3f). These results indicate that rBCG-MyHCα-immunized mice developed DCM and heart failure. 

**Figure 4:**
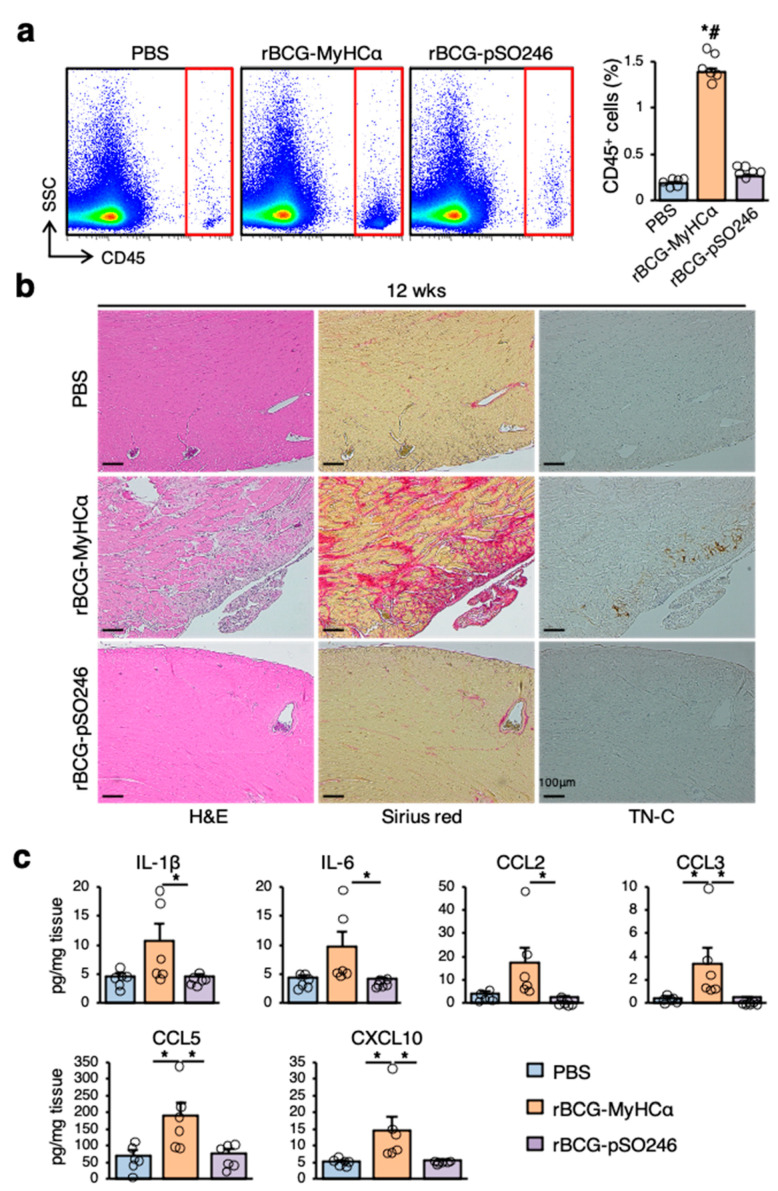
rBCG-MyHCα immunization induced chronic heart inflammation in mice. Immunization protocol is shown in Figure 1a. (**a**) Representative flow cytometric plots showing CD45^+^ leukocyte infiltration in hearts from mice at 12 weeks after rBCG immunization. Bar graph shows the quantification of CD45^+^ leukocytes as a percentage of live cells. Results are presented as mean ± SEM, *n* = 6 each, * *p* < 0.05 vs. PBS and # *p* < 0.05 vs. rBCG-pSO246 by Kruskal-Wallis analysis with a post hoc Steel-Dwass test. (**b**) Representative histology (H&E and Sirius red staining and TN-C immunostaining) of the heart sections at 12 weeks after rBCG immunization. (**c**) Cytokine and chemokine secretion in homogenized hearts obtained from indicated mice at 12 weeks after rBCG immunization was assessed by an ELISA. *n* = 6 per group. * *p* < 0.05 by Kruskal-Wallis analysis with a post hoc Steel-Dwass test.

There was a marked increase in CD45^+^ inflammatory cell infiltration in the hearts of rBCG-MyHCα-immunized mice at 12 weeks after immunization (Figure 4a). Histological examination revealed that larger areas were occupied by Sirius red-positive collagen deposition (Figure 4b). In the fibrotic area, mononuclear cell infiltration was also evident with the expression of TN-C (Figure 4b), and the prevalence of myocarditis was 85.7% (6/7). The heart homogenates from rBCG-MyHCα-immunized mice had significantly increased levels of the proinflammatory cytokines IL-1β and IL-6 and chemokines CCL2, CCL3, CCL5, and CXCL10 (Figure 4c). 

### 2.3. A Model of Prolonged Chronic Myocarditis Following Acute Myocarditis

**Figure 5:**
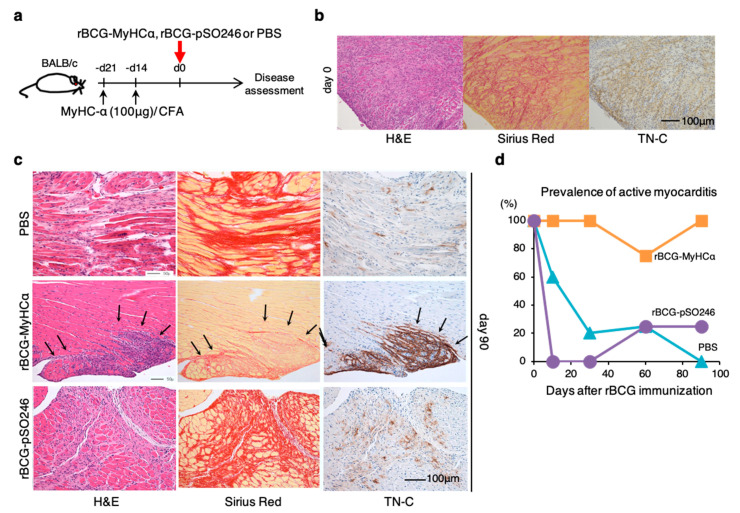
A model of prolonged chronic myocarditis following acute myocarditis. (**a**) Mice were immunized twice with 100 μg of MyHCα peptide/CFA on days 14 and 21 before rBCG immunization to induce EAM. rBCG-MyHCα, rBCG-pSO246, or PBS were injected subcutaneously on day 0. (**b**) Representative histology of the heart sections on day 0 (21 days after first immunization with MyHCα). (**c**) Representative histology of the heart sections at 90 days after rBCG immunization. (**d**) Prevalence of active myocarditis, *n* = 5–6 at each time point for each group.

The clinical picture of chronic myocarditis is thought to be divided into two types: Prolonged chronic myocarditis following acute myocarditis and occult chronic myocarditis with insidious onset. In Figure 3 and Figure 4, mice with rBCG-MyHCα-immunization after priming with a small amount of MyHCα peptide/CFA developed chronic myocarditis without acute myocarditis episode, which can be a model of the latter (occult chronic myocarditis). Therefore, we next tried to establish another model that mimics the former type (prolonged chronic myocarditis following acute myocarditis). We first induced experimental autoimmune myocarditis (EAM) in mice by immunization with 100 μg of MyHCα peptide/CFA before rBCG-MyHCα immunization (Figure 5a) and confirmed that EAM was successfully induced in mice on the day of rBCG immunization (at 3 weeks after the first MyHCα peptide immunization) (Figure 5b). rBCG-MyHCα-immunized EAM mice demonstrated active inflammation even 90 days after rBCG immunization with 100% prevalence (Figure 5c,d). In contrast, in the EAM mice injected with PBS or immunized with rBCG-pSO246, there was almost no active inflammatory cell infiltration or dense TN-C expression in the heart on day 90 (Figure 5c). Based on these findings, rBCG-MyHCα-immunized EAM mice can be a model of prolonged chronic myocarditis following acute myocarditis. 

### 2.4. Assessment of the CD4^+^ T-Cell Function of rBCG-MyHCα-Immunized Mice

**Figure 6:**
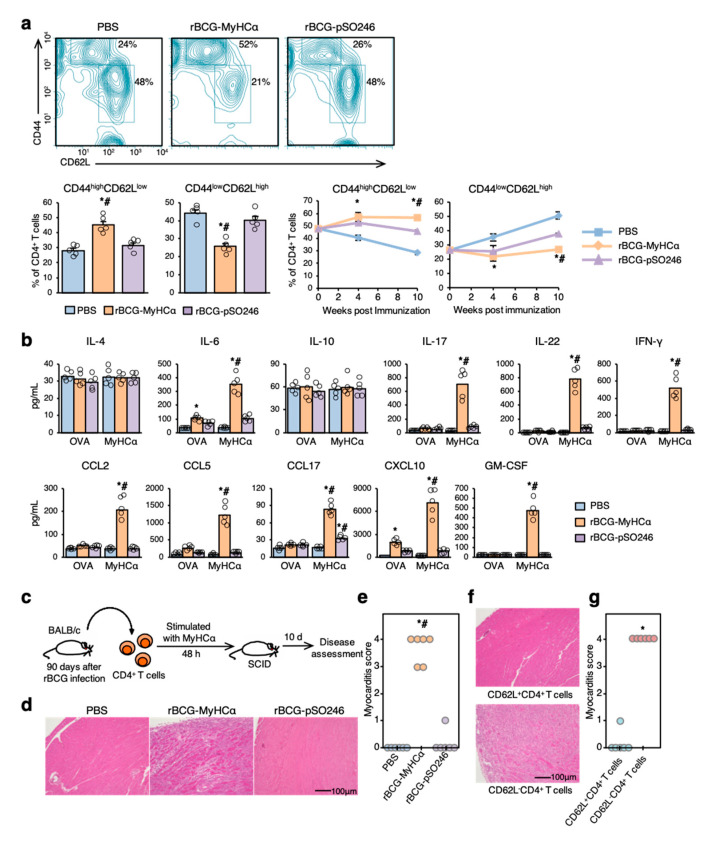
Assessment of CD4^+^ T-cell function. Immunization protocol is shown in Figure 5a. (**a**) Flow cytometric analysis of the heart-infiltrating CD4^+^ T cells at 12 weeks after rBCG immunization. Graphs show the percentage of the effector (CD44^high^CD62L^low^) and naïve (CD44^low^CD62L^high^) T cells in CD4^+^ T cells (*n* = 5 per group). * *p* < 0.05 vs. PBS and # *p* < 0.05 vs. rBCG-pSO246 by Kruskal-Wallis analysis with a post hoc Steel-Dwass test. (**b**) Bar graphs show MyHCα-specific cytokine production of CD4^+^ T cells isolated at 12 weeks after rBCG immunization. *n* = 5 per group. * *p* < 0.05 vs. PBS, # *p* < 0.05 vs. ovalbumin (OVA) stimulation by Kruskal-Wallis analysis with a post hoc Steel-Dwass test. (**c**) CD4^+^ T cells were isolated from rBCG-immunized mice and stimulated with MyHCα for 48 h. After stimulation, the CD4^+^ T cells were transferred into SCID mice. (**d**) Representative H&E-stained section of the hearts at 10 days after adoptive transfer. (**e**) Myocarditis severity in heart sections stained with H&E (*n* = 6 mice/group). * *p* < 0.05 vs. PBS and # *p* < 0.05 vs. rBCG-pSO246. (**f**) CD4^+^ T cells were separated into CD62L^+^ cells and CD62L^−^ cells, and both cell populations were transferred into SCID mice. The representative H&E-stained section of the hearts at 10 days after adoptive transfer (**f**) and the myocarditis score (**g**) are shown. * *p* < 0.05 vs. CD62L^+^CD4^+^ T-cell transfer.

CD4^+^ T cells are key pathogenic players underlying the development and progression of myocarditis, and the effector subsets promote autoimmune responses [22]. Therefore, we next assessed the CD4^+^ T-cell function of rBCG-MyHCα-immunized mice. At 12 weeks after immunization, CD44^high^CD62L^low^ effector memory CD4^+^ T cells were markedly increased, but CD44^low^CD62L^high^ naive CD4^+^ T cells were significantly decreased in the hearts of rBCG-MyHCα-immunized mice (Figure 6a). In the PBS and rBCG-pSO246 groups, the percentage of infiltrating effector memory T cells gradually decreased, while that of naïve T cells increased as myocardial inflammation diminished (Figure 6a). Isolated from rBCG-MyHCα-immunized mice produced proinflammatory cytokines IL-6, IL-17, IL-22, IFN-γ, GM-CSF, and chemokines CCL2, CCL5, CCL17, and CXCL10 in response to MyHCα stimulation (Figure 6b). 

Adoptive transfer of CD4^+^ T cells isolated from rBCG-MyHCα-immunized mice at 12 weeks after immunization successfully induced severe myocarditis in the recipient mice (Figure 6c–e). Moreover, among the CD4^+^ T cells, only CD62L^−^ cells were able to induce myocarditis in the recipients (Figure 6f,g). These results indicate that CD4^+^ T cells from rBCG-MyHCα-immunized mice maintained their effector function in the chronic phase and that CD62L^−^CD4^+^ T cells were the key pathogenic players in this mouse model.

### 2.5. rBCG-MyHCα Mediates DC Activation and Th1/Th17 Cell Differentiation

**Figure 7:**
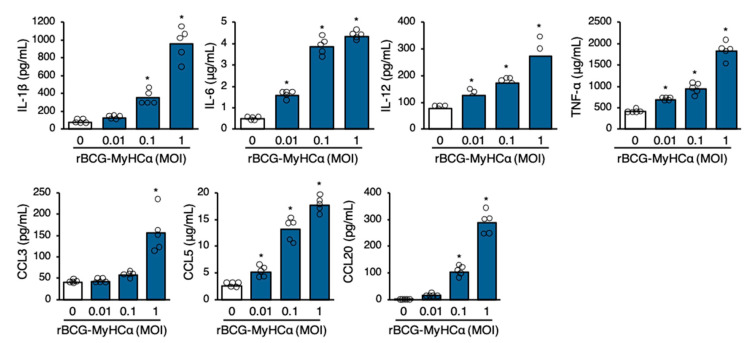
rBCG-MyHCα promoted cytokine and chemokine production in DCs. BMDCs were infected with rBCG-MyHCα at the indicated MOIs for 72 h. Cytokine and chemokine concentrations in the culture supernatant were assessed by ELISA. The values are expressed as means of pentuiplicate wells. * *p* < 0.05 vs. MOI 0 by one-way ANOVA with Tukey’s post hoc test.

DCs are professional APCs that are essential for priming T cell responses [23]. In addition to presenting antigen-derived peptides for T-cell activation and expansion, DCs release a cocktail of polarizing cytokines for the differentiation of CD4^+^ T cells into effector cells [10,24]. rBCG-MyHCα-infected bone marrow-derived DCs (BMDCs) produced proinflammatory cytokines IL-1β, IL-6, IL-12, and TNF-α, and chemokines CCL2, CCL3, CCL5, and CCL20 in an MOI-dependent manner (Figure 7).

**Figure 8:**
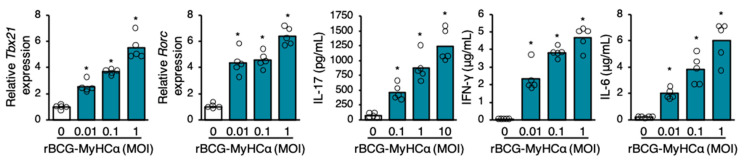
Culture supernatant of rBCG-MyHCα-infected DCs promoted Th1 and Th17 differentiation. Naïve CD4+ T cells were cultured in the supernatant of rBCG-MyHCα-infected BMDCs for 48 h. The gene expression of Tbx21 and Rorc were assessed by qRT-PCR and cytokine concentrations in the culture supernatant were assessed by ELISA. The values are expressed as means of pentuiplicate wells. * *p* < 0.05 vs. MOI 0 by one-way ANOVA with Tukey’s post hoc test.

To investigate the effect of rBCG-MyHCα-infected DCs on CD4^+^ T cell differentiation, naïve CD4^+^ T cells were cultured in the culture supernatant of rBCG-MyHCα-infected DCs. The transcription factors, T-bet (encoded by Tbx21) and RORγT (encoded by Rorc), which have critical roles in the development of Th1 and Th17 cells, respectively, were upregulated, and the CD4^+^ T cells produced Th1 and Th17 cytokines (IFN-γ and IL-17, respectively) in an MOI-dependent manner (Figure 8). Thus, rBCG-MyHCα infection encouraged the differentiation of CD4^+^ T cells into Th1 and Th17 cells via DC activation. 

### 2.6. rBCG-Infected DCs Induce MyHCα-Specific CD4^+^ T-Cell Proliferation

**Figure 9:**
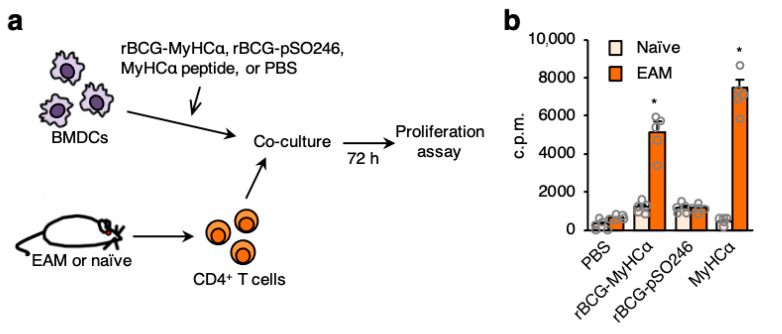
rBCG-MyHCα-infected DCs induced MyHCα-specific CD4^+^ T-cell proliferation. (**a**) EAM was induced by immunization with MyHCα peptide emulsified in CFA. CD4^+^ T cells were isolated from spleens of EAM or naïve mice and co-cultured with rBCG-infected BMDCs, MyHCα-loaded BMDCs, or non-stimulated BMDCs (PBS). (**b**) T-cell proliferation was assessed by measurement of [^3^H]-thymidine incorporation. The values are expressed as means ± SEM of pentuiplicate wells. * *p* < 0.05 vs. naive by one-way ANOVA with Tukey’s post hoc test.

Next, we investigated whether DCs infected with rBCG-MyHCα could activate MyHCα-specific T cells. MyHCα-specific CD4^+^ T cells were isolated from EAM mice and co-cultured with DCs (Figure 9a). MyHCα-specific CD4^+^ T cells significantly proliferated when co-cultured with rBCG-MyHCα-infected DCs or with MyHCα peptide-loaded DCs (Figure 9b). These results indicated that DCs infected with rBCG-MyHCα presented MyHCα peptide to CD4^+^ T cells and activated them.

### 2.7. rBCG-Infected DCs Can Induce Chronic Myocarditis

**Figure 10:**
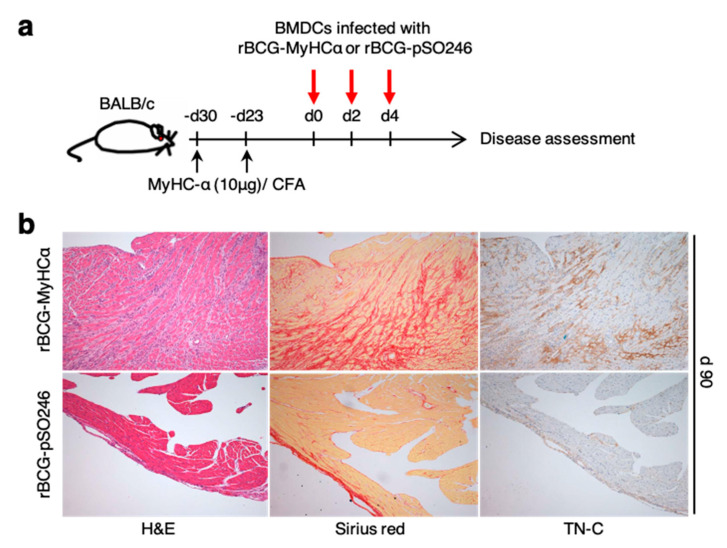
rBCG-MyHCα-infected DCs induced chronic myocarditis. (**a**) After priming with 10 μg of MyHCα peptides on days-30 and -23, BMDCs infected with rBCG-MyHCα or rBCG-pSO246 were injected into mice on days 0, 2, and 4. (**b**) Representative histology (H&E and Sirius red staining and TN-C immunostaining) of the heart sections on 90 days after DC injection.

Finally, we investigated whether rBCG-infected DCs could induce chronic myocarditis. After priming with a small amount of MyHCα peptides, BMDCs infected with rBCG-MyHCα or rBCG-pSO246 were injected into mice (Figure 10a). Histological examination revealed severe mononuclear cell infiltration with collagen deposition and TN-C expression on day 90 after rBCG-MyHCα-infected BMDC injection (Figure 10b). These results suggest that rBCG-MyHCα-infected DCs continuously activated MyHCα-reactive CD4^+^ T cells that induced chronic myocarditis.

## 3. Discussion

In this study, we established a novel mouse model of chronic myocarditis induced by recombinant BCG expressing a CD4^+^ T-cell epitope of cardiac myosin. Mice immunized with rBCG-MyHCα after priming with a low dose of MyHCα peptide/CFA mimicked occult chronic myocarditis in humans; rBCG-MyHCα immunization after EAM induction represented prolonged chronic myocarditis following acute myocarditis. CD44^high^CD62L^low^CD4^+^ cells were increased in the heart and showed Th1/Th17 phenotypes. Adoptive transfer of CD4^+^ T cells induced myocarditis in the recipients even 12 weeks after rBCG-MyHCα immunization, which indicated that rBCG-MyHCα maintained the effector function of CD4^+^ T cells for extended periods of time. 

This is the first report of the successful establishment of an autoimmune disease model using rBCG technology. BCG offers some unique advantages for developing chronic autoimmune responses against recombinant autoantigens. BCG can persist in vivo for many years, which could provide continuing immunization to the recombinant autoantigen. In addition, it is well known that mycobacterial components are typical immuno-adjuvants, such as the CFA, which is used for the induction of EAM. The cell wall components of BCG induce innate immunity via toll-like receptors (TLRs) 2 and 4 on DCs and macrophages [25]. DNA fragments containing the CpG motif also activate innate immunity via the TLR9 pathway [26]. TLR signaling pathways culminate in the activation of the transcription factor nuclear factor-kappaB, triggering the secretion of a broad range of inflammatory cytokines [27]. Moreover, due to the internalization of BCG or phagocytosis, APCs process BCG and present antigens to CD4^+^ T cells by binding with major histocompatibility complex class II molecules to activate them. After APC presents the antigen to T cells, CD4^+^ T cells release cytokines, including Th1 and Th17 cytokines [28]. In our study, rBCG-MyHCα-infected DCs produced proinflammatory cytokines and induced MyHCα-specific T-cell proliferation and Th1 and Th17 polarization. Thus, BCG may be an ideal vehicle for the delivery of autoantigens to induce chronic autoimmune responses.

The Japanese Circulation Society Task Force Committee on Chronic Myocarditis proposed two types of chronic myocarditis: Prolonged chronic myocarditis following acute myocarditis and occult chronic myocarditis with insidious onset [29]. The clinical picture of the two types of chronic myocarditis appears to differ considerably. The former often changes from acute fulminant myocarditis with/after mechanical circulatory support, while the latter type is usually incidentally diagnosed by endomyocardial biopsy in DCM patients. In either type, prolonged inflammation causes progressive tissue destruction, eventually resulting in DCM [3]. In this study, we established mouse models of these two types of chronic myocarditis. EAM mice with rBCG-MyHCα-immunization can be a model of prolonged chronic myocarditis following acute myocarditis. On the other hand, mice with rBCG-MyHCα-immunization after priming with a small amount of MyHCα peptide/CFA developed chronic myocarditis without acute myocarditis episode, which can be a model of occult chronic myocarditis. 

In our chronic myocarditis model, CD4^+^ T cells produced large amounts of IFN-γ and IL-17 in response to MyHCα. The importance of MyHCα-specific Th1 and Th17 cells in the development of autoimmune myocarditis has been demonstrated in a number of animal studies. Mice lacking T-bet, a transcription factor that is essential for Th1 differentiation and IFN-γ production, were highly susceptible to autoimmune myocarditis owing to the induction of IL-17 production [30]. In EAM mice, Th17 cells promoted the progression of cardiac dysfunction [31]. In a transgenic mouse model expressing a MyHCα-specific T cell receptor on CD4^+^ T cells, myocarditis developed spontaneously, and the cooperation of IFN-γ and IL-17A was found to be essential for the transition from myocarditis to DCM [32]. The association between Th1 and Th17 immune responses and disease progression has also been suggested in human myocarditis/DCM. Peripheral blood T cells from myocarditis patients exhibited significantly higher IFN-γ reactivity against the MyHCα peptide when compared with healthy controls. Moreover, myocarditis/DCM patients with severe heart failure have greater proportions of Th17 than those with low severity heart failure [33]. In addition to IFN-γ and IL-17, our model had cytokine profiles similar to those of myocarditis patients, including elevated levels of IL-6 and GM-CSF [33]. Thus, this novel chronic myocarditis mouse model may allow the identification of the central pathophysiological and immunological processes involved in the progression to DCM.

## 4. Materials and Methods 

### 4.1. Animals

BALB/c mice and CB17.SCID mice were purchased from CLEA Japan. We used 7- to 9-week-old male mice. All animal experiments were approved by the Institutional Animal Experiment Committee of the National Institute of Biomedical Innovation, Health and Nutrition, Tsukuba, Japan (approved number: 11328, approved date: 1 December 2008) and conformed to the NIH Guide for the Care and Use of Laboratory Animals.

### 4.2. Preparation of rBCG

Oligonucleotides that encode a 16-amino-acid MyHCα CD4^+^ T-cell epitope (aa 614–629; SLKLMATLFSTYASAD) were chemically synthesized. The sequences were 5′-TCGAGTCTGAAGCTGATGGCGACCCTGTTCTCGACCTACGCGTCGGCGGAT-3′ for the upper strand and 5′-TCGAATCCGCCGACGCGTAGGTCGAGAACAGGGTCGCCATCAGCTTCAGAC-3′ for the lower strand with cohesive ends of the XhoI recognition site (underlined) at both terminals of each DNA. The DNA oligomers were cloned into an XhoI site in the coding region of the Ag85B gene from *M. kansasii* cloned into pSO246 to be fused with the gene, and the generated plasmid was named pSO246-MyHCα. pSO246-MyHCα and pSO246 (empty plasmid: control) vectors were introduced into *M. bovis* BCG Tokyo 172 strain by electroporation, giving rise to rBCG-MyHCα and rBCG-pSO246, respectively. These transformants were selected on a Difco Middlebrook 7H10-agar (BD) plates containing Middlebrook OADC enrichment (BD) and 30 μg/mL kanamycin and grown in Difco Middlebrook 7H9-broth (BD) containing Middlebrook ADC enrichment (BD) and 0.05 Tween 80 at 37 °C for 2 weeks. After harvesting, rBCG was washed twice with PBS and then used for immunization.

### 4.3. Western Blot Analysis

The BCG cell lysates and a portion of the culture supernatant were subjected to SDS-PAGE using a 4%–20% gradient gel. The fractionated proteins were electroblotted onto a nitrocellulose membrane filter, reacted with rabbit anti-Ag85B polyclonal antibodies that had been absorbed with the cross-reactive Ag85B from BCG and reacted with peroxidase-conjugated goat anti-rabbit IgG, and then visualized with a substrate (3,3′-diaminobenzidine) of peroxidase.

### 4.4. rBCG Immunization Protocols

A total of 500 μg of rBCG was injected s.c. into mice. In some experiments, mice were immunized twice with 10 μg of the MyHCα peptide (MyHCα_614–629_) Ac-RSLKLMATLFSTYASADR-OH (Toray Research Center) emulsified 1:1 in PBS/CFA (1 mg/mL; H37Ra; Sigma-Aldrich, St. Louis, MI, USA) as described previously [12,34,35] 14 and 21 days prior to the rBCG injection. For rBCG-infected-DC immunization, BMDCs were generated as previously described [36]. BMDCs were infected with rBCG at a multiplicity of infection of 1:5. After infection, cells were allowed to phagocytose for 4 h, and any nonphagocytosed bacteria were cleared by washing with PBS, and then incubated for 24 h. Recipient mice were injected with 5 × 10^5^ rBCG-infected BMDCs i.p. on days 0, 2, and 4.

### 4.5. Histopathological and Immunohistochemical Examination

The hearts were fixed in 4% paraformaldehyde in PBS and embedded in paraffin wax. For histological analysis, 3-μm-thick sections were cut and stained with H&E and Sirius Red. To evaluate the expression of TN-C, we performed immunohistochemistry as previously described [37]. In brief, sections after antigen retrieval were incubated with polyclonal rabbit anti-TN-C antibodies [38,39], followed by treatment with HRP-conjugated goat anti-rabbit antibody (MBL, Nagoya, Japan). The antibody reactions were visualized using diaminobenzidine chromogen and counterstained with hematoxylin.

### 4.6. Echocardiography

Transthoracic echocardiography was performed using a Prosound α6 with a 10-MHz transducer (Aloka). The diastolic (LVDd) and systolic left ventricular (LV) dimensions (LVDs) were measured from the M-mode tracings. LV fractional shortening (FS), a measure of LV systolic function, was calculated from the M-mode LV dimensions using the following equation: FS (%) = [(LVDd − LVDs)/LVDd] × 100. Two independent investigators who conducted the echocardiography were unaware of the immunization status.

### 4.7. Hemodynamic Assessment

Hemodynamic assessment was performed as previously described [13]. Briefly, the mice were anesthetized with sodium pentobarbital (50 mg/kg), and the LV apex was exposed via a subdiaphragmatic incision. An apical stab was made with a 27-gauge needle, containing a fiber pressure sensor (FPI-LS-PT9; FISO Technology Inc., QC), placed to span the long axis of the LV. All signals were analyzed with a signal conditioner (FPI-LS-10; FISO Technology Inc., Québec, QC, Canada) and data acquisition system (LabTrax-4) and then stored on disks for off-line analysis using a software (LabScribe). The following indices were assessed: heart rate, systolic LV pressure, and maximal and minimum rates of LV pressure development (±dP/dt).

### 4.8. Flow Cytometric Analyses

For the flow cytometric analysis of surface markers and cytoplasmic cytokines, cells were stained directly using fluorochrome-conjugated mouse-specific antibodies and analyzed with a FACSCalibur instrument (BD Biosciences). Antibodies were purchased from eBioscience, including CD4, CD44, CD45, and CD62L.

### 4.9. Measurements of Cytokines and Chemokines

Hearts were homogenized in media containing 2.5% FBS. Supernatants were collected after centrifugation and stored at −80 °C. To investigate the MyHCα-specific T-cell response, CD4^+^ T cells were isolated from the splenocytes using magnetic-activated cell sorting kits (CD4 [L3T4] MicroBeads, Miltenyi Biotec) and stimulated with 5 μg/mL of MyHCα or ovalbumin (OVA) peptide in the presence of APCs for 48 h. Concentrations of cytokines and chemokines in the heart homogenates or culture supernatants were measured with Quantikine ELISA kits (R&D Systems, Minneapolis, MN, USA).

### 4.10. Adoptive Transfer of T Cells

Splenocytes were collected from immunized mice and cultured with 5 μg/mL MyHCα for 48 h. Briefly, 5 × 10^6^ CD4^+^ T cells were purified using anti-CD4 magnetic beads (Miltenyi Biotec) and injected i.p. into the SCID mice. The mice were killed 10 days after injection for further analysis.

### 4.11. RNA Extraction and Quantitative Real-Time RT-PCR

Total RNA was extracted from cells using the MagNA Pure Compact Instrument (Roche Applied Science, Penzberg, Germany) together with MagNA Pure Compact RNA Isolation Kit (Roche Applied Science) according to the manufacturer’s instructions. cDNA was synthesized from 1 μg total RNA using an Omniscript RT kit (Qiagen, Hilden, Germany). qRT-PCR analysis was performed on the LightCycler 480 system (Roche Applied Science) with the Universal Probe Library (Roche Applied Science). The primers used for the PCR amplification were as follows: *Tbx21* forward, TCAACCAGCACCAGACAGAG; *Tbx21* reverse, AAACATCCTGTAATGGCTTGTG; *Rorc* forward, CCCTGGTTCTCATCAATGC; *Rorc* reverse, TCCAAATTGTATTGCAGATGTTC; *Hprt* forward, TCCTCCTCAGACCGCTTTT; and *Hprt* reverse CCTGGTTCATCATCGCTAATC. The data were normalized by the level of *Hprt* expression in each sample. 

### 4.12. In Vivo Experiments of rBCG-MyHCα-Infected DCs 

BMDCs were cultured in 12-well flat-bottomed cell culture plates (1 × 10^6^/mL) and infected with rBCG-MyHCα at MOI of 0.01, 0.1, or 1 for 72 h at 37 °C in a humidified atmosphere containing 5% CO_2_. 

### 4.13. Naïve CD4^+^ T-Cell Isolation

Naïve CD4^+^ T cells were isolated from the splenocytes of BALB/c mice using a magnetic-activated cell sorting kit (CD4^+^CD62L^+^ T Cell Isolation Kit II, Miltenyi Biotec). 

### 4.14. Proliferative Responses of T Cells

T-cell proliferation was assessed as previously described [12]. To obtain MyHCα-reactive CD4^+^ T cells, EAM mice were immunized with 100 μg of the MyHCα peptide emulsified 1:1 in PBS/complete Freund’s adjuvant (CFA) on days 0 and 7, and the spleens were collected on day 14. CD4^+^ T cells were isolated from splenocytes using magnetic-activated cell sorting kits (CD4 [L3T4] MicroBeads, Miltenyi Biotec, Bergisch Gladbach, Germany). Naïve CD4^+^ T cells were isolated from BALB/c mice as described above. CD4^+^ T cells were co-cultured with rBCG-MyHCα-infected BMDCs, rBCG-pSO246-infected BMDCs (negative control), 5 μg/mL MyHCα-loaded BMDCs (positive control), or non-treated BMDCs (negative control) for 72 h and pulsed with 0.5 μCi of [^3^H]-thymidine 8 h before being measured with a beta counter. 

### 4.15. Statistical Analysis

All data are expressed as mean ± SEM. Normality was verified using the Shapiro-Wilk test. Statistical analyses were performed using an unpaired two-tailed *t* test or Mann-Whitney *U* test for comparison of two groups. For multiple comparisons, one-way analysis of variance (ANOVA) with a Tukey post hoc test or a Kruskal-Wallis analysis with a post hoc Steel-Dwass or Steel test was used. A *p* < 0.05 was considered statistically significant. All statistical analyses were performed using JMP software (SAS Institute, Cary, NC, USA).

## Data Availability

The data that support the findings of this study are available from the corresponding author (Y.Y.), upon reasonable request.

## Abbreviations

APC	antigen-presenting cell
BCG	bacillus Calmette-Guérin
CFA	complete Freund’s adjuvant
DC	dendritic cell
DCM	dilated cardiomyopathy
+dP/dt	maximum rates of left ventricular pressure development
−dP/dt	minimum rates of left ventricular pressure development
EAM	experimental autoimmune myocarditis
FS	fractional shortening
H&E	hematoxylin and eosin
LV	left ventricular
LVDd	left ventricular diastolic dimension
LVDs	left ventricular systolic dimension
MyHCα	myosin heavy chain-α
rBCG	recombinant bacillus Calmette-Guérin
rBCG-MyHCα	rBCG expressing a CD4^+^ T-cell epitope of myosin heavy chain-α
rBCG-pSO246	empty plasmid (pSO246)-electroporated rBCG
Th	T helper
TN-C	tenascin-C
